# An optimized method for the extraction of bacterial *m*RNA from plant roots infected with *Escherichia coli* O157:H7

**DOI:** 10.3389/fmicb.2014.00286

**Published:** 2014-06-10

**Authors:** Ashleigh Holmes, Louise Birse, Robert W. Jackson, Nicola J. Holden

**Affiliations:** ^1^Cell and Molecular Sciences, The James Hutton InstituteInvergowrie, Dundee, UK; ^2^School of Biological Sciences, The University of ReadingKnight Building, Whiteknights, Reading, UK

**Keywords:** food-borne pathogens, lettuce, spinach, rhizosphere, *m*RNA isolation

## Abstract

Analysis of microbial gene expression during host colonization provides valuable information on the nature of interaction, beneficial or pathogenic, and the adaptive processes involved. Isolation of bacterial *m*RNA for *in planta* analysis can be challenging where host nucleic acid may dominate the preparation, or inhibitory compounds affect downstream analysis, e.g., quantitative reverse transcriptase PCR (qPCR), microarray, or RNA-seq. The goal of this work was to optimize the isolation of bacterial *m*RNA of food-borne pathogens from living plants. Reported methods for recovery of phytopathogen-infected plant material, using hot phenol extraction and high concentration of bacterial inoculation or large amounts of infected tissues, were found to be inappropriate for plant roots inoculated with *Escherichia coli* O157:H7. The bacterial RNA yields were too low and increased plant material resulted in a dominance of plant RNA in the sample. To improve the yield of bacterial RNA and reduce the number of plants required, an optimized method was developed which combines bead beating with directed bacterial lysis using SDS and lysozyme. Inhibitory plant compounds, such as phenolics and polysaccharides, were counteracted with the addition of high-molecular-weight polyethylene glycol and hexadecyltrimethyl ammonium bromide. The new method increased the total yield of bacterial *m*RNA substantially and allowed assessment of gene expression by qPCR. This method can be applied to other bacterial species associated with plant roots, and also in the wider context of food safety.

## INTRODUCTION

The analysis of bacterial gene expression is important in the determination of how adaptation to different environments develops and informs on the roles of different genes during this process. Human pathogens are now recognized to interact with plants and use them as hosts, as a result of recent high-profile outbreaks from contaminated fruit and vegetables (Cooley et al., 2007; Buchholz et al., 2011). Adaptation of food-borne pathogens to secondary hosts has opened up new areas of research and investigation. Current research suggests that the interaction of human pathogens is more complex than previously perceived in that they can persist for long periods of time (reviewed in Brandl, 2006; Holden et al., 2009; Barak and Schroeder, 2012) and invoke an immune response from the plant (Thilmony et al., 2006; Schikora et al., 2008; Roy et al., 2013). Furthermore, the interactions include quite specific and targeted recognition of the plant host cells by the bacteria (Rossez et al., 2013). Important questions remain as to how the bacteria adapt to secondary hosts, for which analysis of bacterial gene expression is fundamental.

Bacterial gene expression is typically assessed using quantitative reverse transcriptase PCR (qPCR) or microarray techniques, and more recently RNA-seq (An et al., 2013), which require the isolation of high quality and quantity of *m*RNA. Bacterial *m*RNA has a short half-life and is less stable than eukaryotic *m*RNA transcripts, which have capped and polyadenylated RNA tails. Therefore, appropriate measures need to be in place to capture *m*RNA transcripts that may be inherently unstable, but nevertheless important for bacterial adaptation. In addition to challenges with bacterial *m*RNA stability, extractions from mixed samples bring additional considerations, not least for inhibitory compounds that may affect downstream analysis. Although there are a number of published techniques for the isolation of total RNA from bacteria-infected plant leaves (Schenk et al., 2008; Soto-Suarez et al., 2010; Fink et al., 2012; Goudeau et al., 2013), samples can still be dominated by plant RNA making it challenging to assess bacterial *m*RNA. In published reports, leaves were typically infected via infiltration, introducing a high bacterial inoculum into the sample. In other studies, total RNA was extracted from inoculated plant extracts (Mark et al., 2005; Hernandez-Morales et al., 2009; Kyle et al., 2010; Shidore et al., 2012), such as leaf lysates, which helped to reduce the dominance of plant RNA in the sample.

There are limited studies where bacterial expression has been assessed directly from the plant root system (roots and rhizosphere). However, roots are known to support relatively high densities of bacteria, providing a more favorable habitat than the phyllosphere. In general, published reports describe that a high number of plant roots is required to retrieve sufficient bacterial RNA (Matilla et al., 2007; Hou et al., 2012, 2013; Zyśko et al., 2012). Further to this, the application of techniques described for RNA purification from infected leaves cannot always be successfully applied to roots. Therefore, optimization of the RNA extraction methods is required to obtain sufficient quantity and quality of bacterial *m*RNA, coupled with a reduction in the amount of accompanying plant root RNA. We optimized the method for the food-borne pathogen *Escherichia coli* O157:H7, frequently associated food-borne outbreaks from consumption of contaminated spinach and lettuce. Although roots of these plants are not consumed, the pathogen can colonize this habitat successfully (Wright et al., 2013), from where it can contaminate the edible portion, either directly or through cross-contamination during processing.

## MATERIALS AND METHODS

### BACTERIAL STRAINS AND GROWTH CONDITIONS

*E. coli* O157:H7 isolate Sakai *stx*-negative (Hayashi et al., 2001) was routinely cultured overnight in Luria broth at 37°C, with aeration. For plant-infection assays, the bacteria were sub-cultured at 1:50 dilution into 15 ml rich-defined MOPs media supplemented with 0.2% glucose (RD MOPS glucose; Neidhardt et al., 1974), in a 200 ml Erlenmeyer flask and incubated at 18°C, with aeration for ~18 h. Bacterial cultures were diluted to OD_600_ of 0.2 (equivalent to ~2 × 10^8^ cfu ml^-^^1^) in sterile phosphate buffered saline (PBS) prior to infecting plant roots.

### PLANT PROPAGATION AND INFECTION

Lettuce (*Lactuca sativa*) cultivar All Year Round or spinach (*Spinacia oleracea*) cultivar Amazon seeds (Sutton Seeds, UK) were soaked in sterile distilled water for 2 h before being surface sterilized in 2% calcium hypochlorite solution (10 ml) for 10 min. The seeds were then washed vigorously six times with sterile distilled water and germinated on distilled water agar (0.5% w/v) in the dark for 3–5 days, at ~22°C. Seedlings were transplanted into 175 ml hydroponic tubes (Greiner, Frickenhausen, Germany) containing autoclaved perlite and sterile 0.5× Murashige and Skoog (MS) medium (Sigma Aldrich, USA). Seedlings were grown in a cabinet with a light intensity of 150 μmol m^2^ s^-^^1^ (16 h photoperiod) for a further 21 days at 22°C. To assess bacterial numbers from infected plant material, the roots were washed with PBS to remove excess, non-adherent bacteria. The root sample was homogenized using mortar and pestle and serially diluted for viable counts on selective agar plates.

### RNA EXTRACTION AND PURIFICATION

**FIGURE 1:**
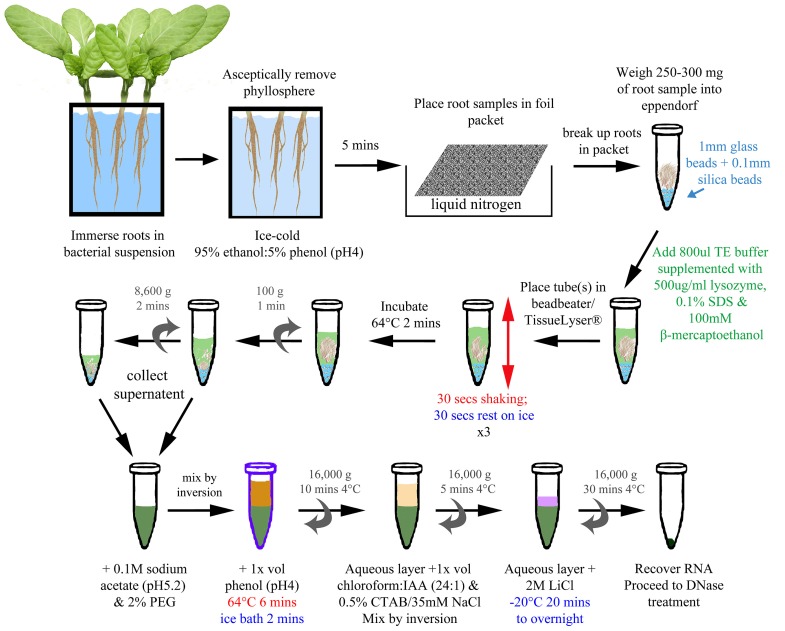
**Bead/SDS/phenol method of RNA extraction from infected plant roots**. A schematic of the optimized method (described in detail within Section “Material and Methods”). The final concentrations of reagents are indicated in the figure. Colored text has been used to highlight different stages and treatments (e.g., blue for addition of beads/cold; green for lysis; red for shaking/heat).

Total RNA was extracted from infected root samples either by the BPEX method (Schenk et al., 2008) or by the Bead/SDS/phenol method (**Figure 1**): a method which was adapted and optimized from Jahn et al. (2008) and Schenk et al. (2008).

#### Sample preparation

Sample preparation was common to both methods. Whole plants were gently removed from the hydroponic tubes and washed with sterile PBS to remove as much perlite as possible before bacterial infection. Plants were pooled into groups of 5 and the roots were submerged into 20–25 ml bacterial suspension (OD_600_ = 0.2 equivalent to ~ 5 × 10^7^–1 × 10^8^ cfu ml^-^^1^), and incubated at 18°C for 2 h. After incubation, the plants were removed from the bacterial suspension and all the tissue above the crown (shoots, leaves, and petioles) were aseptically removed with a sterile scalpel and the roots immediately immersed into 20 ml ice-cold, 95% ethanol: 5% phenol (pH 4.0). The sample was incubated on ice for 5 min and gently agitated on a vortex mixer to remove any excess and loosely attached bacteria. The root sample was placed into a foil packet and immediately stored in liquid nitrogen until all samples had been processed.

#### BPEX-Schenk method for extraction

The BPEX method was originally developed to recover *Pseudomonas syringae* phytopathogen *m*RNA from infected plant material (Schenk et al., 2008), and tested for recovery of *E. coli m*RNA from infected roots. The main change from the published method was in the tissue type (root vs leaf disks), although the protocol is provided to allow a direct comparison with the optimized method. Infected plant tissue was ground to a fine powder in liquid nitrogen with a pre-cooled pestle and mortar. The sample (~1 g) was transferred to an Eppendorf containing 750 μl of *b*acteria *p*lant *ex*traction (BPEX) buffer [0.35 M glycine, 0.7 M NaCl, 2% (w/v) polyethylene glycol (PEG) 20000, 40 mM EDTA, 50 mM NaOH, 4% (w/v) SDS] supplemented with 100 mM β-mercaptoethanol just prior to use. The sample was mixed on a vortex mixer in the buffer prior to incubation at 95°C for 90 s with shaking. An equal volume (750 μl) of phenol/chloroform mix (5:1, pH 4.0) (Sigma, St. Louis, USA) was added and the sample shaken for 5 min to form an emulsion before centrifugation at 16,000 × *g* for 7 min. The upper phase was collected and added to an equal volume of phenol/chloroform mix (5:1, pH 4.0), shaken, and centrifuged again as the previous step. The upper phase was collected and added to an equal volume of phenol/chloroform/isoamyl alcohol (IAA) mix (25:24:1, pH 4.0) (Sigma, St. Louis, USA) shaken and centrifuged again as before. A volume of 495 μl of the upper phase was added to 550 μl chloroform/IAA mix (24:1) and overlayed with 55 μl pre-warmed (55°C) hexadecyltrimethyl ammonium bromide (CTAB)/NaCl (10% CTAB, 0.7 M NaCl) solution. The suspension was shaken and centrifuged as before. The upper phase was added to 1/4 vol 8 M LiCl solution, mixed by inversion and the RNA precipitated at –20°C for 30 min. To collect the RNA, the sample was centrifuged at 16,000 × *g* for 20 min at 4°C and the RNA pellet resuspended in 100 μl RNase-free water. The RNA was cleaned and DNase treated using RNeasy Plant Mini kit (Qiagen, Limburg, Netherlands) as per the manufacturer’s guidelines.

#### Bead/SDS/phenol method for extraction

The optimized method is represented in **Figure 1**. The samples were removed from the liquid nitrogen, beaten with a spatula to break up the root into smaller pieces, and transferred into an 1.5 ml micro-centrifuge tube (DNase, RNase free: Ambion, Austin, USA) preloaded with ~250 mg mixture of 1 mm glass and 0.1 mm silica beads (ThermoScientific Ltd., Waltham, USA), from which the weight was determined. Cooled, sterile equipment was used throughout the process. The micro-centrifuge tube was then returned to liquid nitrogen for processing subsequent samples until all of the samples were collected. For the lysis step, 800 μl of Tris-EDTA (TE) buffer (10 mM Tris-HCl; 1 mM EDTA) supplemented with 500 μg ml^-^^1^ lysozyme, 0.1% SDS, and 100 mM β-mercaptoethanol was added to each root sample. The tubes were placed into TissueLyser (Qiagen, Limburg, Netherlands) and agitated for 30 s with a 30 s interval on ice for three cycles. After the last cycle, the samples were returned to ice before transfer to a heatblock set at 64° C for 2 min. To extract nucleic acids, the supernatant was collected and pooled after two centrifugation steps: first at 100 × *g* for 1 min to pellet the beads and large fragments of root, followed by a second at 8,600 × *g* for 2 min to compact the debris further, yielding more supernatant. One hundred millimolar sodium acetate (pH 5.2) and 2% (w/v) PEG 20000 was added to the supernatant and inverted to mix. An equal volume (~1 ml) of phenol (pH 4.0) was then added, mixed by inversion, and the sample incubated at 64°C for 6 min, with the tubes inverted every 40 s. The sample was transferred to an ice bath for 2 min before centrifugation at 16,000 × *g* for 10 min at 4°C. The upper aqueous layer was added to an equal volume of chloroform/IAA mix (24:1) and 1/20 volume of pre-warmed (55°C) CTAB/NaCl solution in a fresh micro-centrifuge tube. The sample was mixed by inversion and then centrifuged at 16,000 × *g* for 5 min at 4°C. The upper aqueous layer was added to 1/4 vol 8 M LiCl, mixed by inversion, and then incubated at –20°C for 20 min to overnight to precipitate the nucleic acid. The nucleic acid was recovered by centrifugation at 16,000 × *g* for 30 min at 4°C. The pellet was resuspended in 100 μl RNase-free water and the sample cleaned and DNase treated using the RNeasy Plant Mini kit (Qiagen, Limburg, Netherlands) as per the manufacturer’s instructions.

Total RNA concentration was determined using a NanoDrop (Wilmington, USA) spectrophotometer and the relative proportions of ribosomal RNA determined using a BioAnalyser 2100 (Agilent Technologies, Santa Clara, USA), for both methods.

#### *c*DNA Synthesis and qPCR Conditions

*c*DNA was transcribed from 1 μg total RNA using Superscript II (Invitrogen, Carlsbad, USA) following the random primer protocol. A mixture of ten 11-mer oligonucleotide primers (Ea1 ± Ea10) at 100 nM (Fislage et al., 1997) designed specifically for *Enterobacteriaceae m*RNA was used. Quantitative reverse transcriptase PCR was carried out using specific primers for *E. coli* O157:H7 *gyrB* housekeeping gene (*gyrB*.RT.F = CATCAGAGAGGTCGGCTTCC; *gyrB*.RT.R = CATGGAGCGTCGTTATCCGA) using StepOnePlus^TM^ real-time PCR system (Applied Biosystems, Life Technologies, Carlsbad, USA) and iTaq^TM^ Universal SYBR® Green Supermix (Bio-Rad, Hercules, USA). Each 20 μl reaction volume was composed of iTaq SuperMix, 300 nM of forward and reverse primers and 1 μl of *c*DNA (diluted 1:4). The PCR program consisted of an initial denaturation at 95°C for 10 min, followed by 40 cycles of denaturation at 95°C for 15 s, and annealing and extension at 60°C for 1 min. Melt curve analysis was also performed with an initial denaturation at 95°C for 15 s, followed by annealing at 60°C for 1 min with an 0.3°C increase (step and hold) and final step of 95°C for 15 s. Data were collected from three technical replicates and from two biological replicates.

## RESULTS

### EXTRACTION OF TOTAL RNA FROM PLANT ROOTS INFECTED WITH BACTERIA

An optimized method was previously reported for the extraction of *P. syringae m*RNA from infiltrated plant leaves (Schenk et al., 2008). A buffer system was developed that yielded 75–125 μg of total RNA per 150–200 mg sample, from the equivalent of 20 infiltrated leaf disks (7 mm). We tested this protocol (termed the “BPEX” method) to determine if it could also be used to recover *m*RNA of a food-borne pathogen from infected roots. Our work focuses on the bacterial genes induced during the stages of colonization of lettuce and spinach roots, and as such the roots in our experiments are not infected via infiltration; rather the bacteria colonize the outer surface. This necessitated processing the entire root to recover sufficient RNA. Also, to obtain equivalent bacterial numbers to those reported, where each leaf disk was infiltrated with 1 × 10^8^ cfu bacteria, at least 20 root samples were pooled for each extraction. In this method, the samples were ground to a fine powder in liquid nitrogen, prior to incubation in an extraction buffer (BPEX) coupled with cell lysis. RNA was purified using traditional acidic phenol extraction and LiCl-mediated precipitation.

**FIGURE 2:**
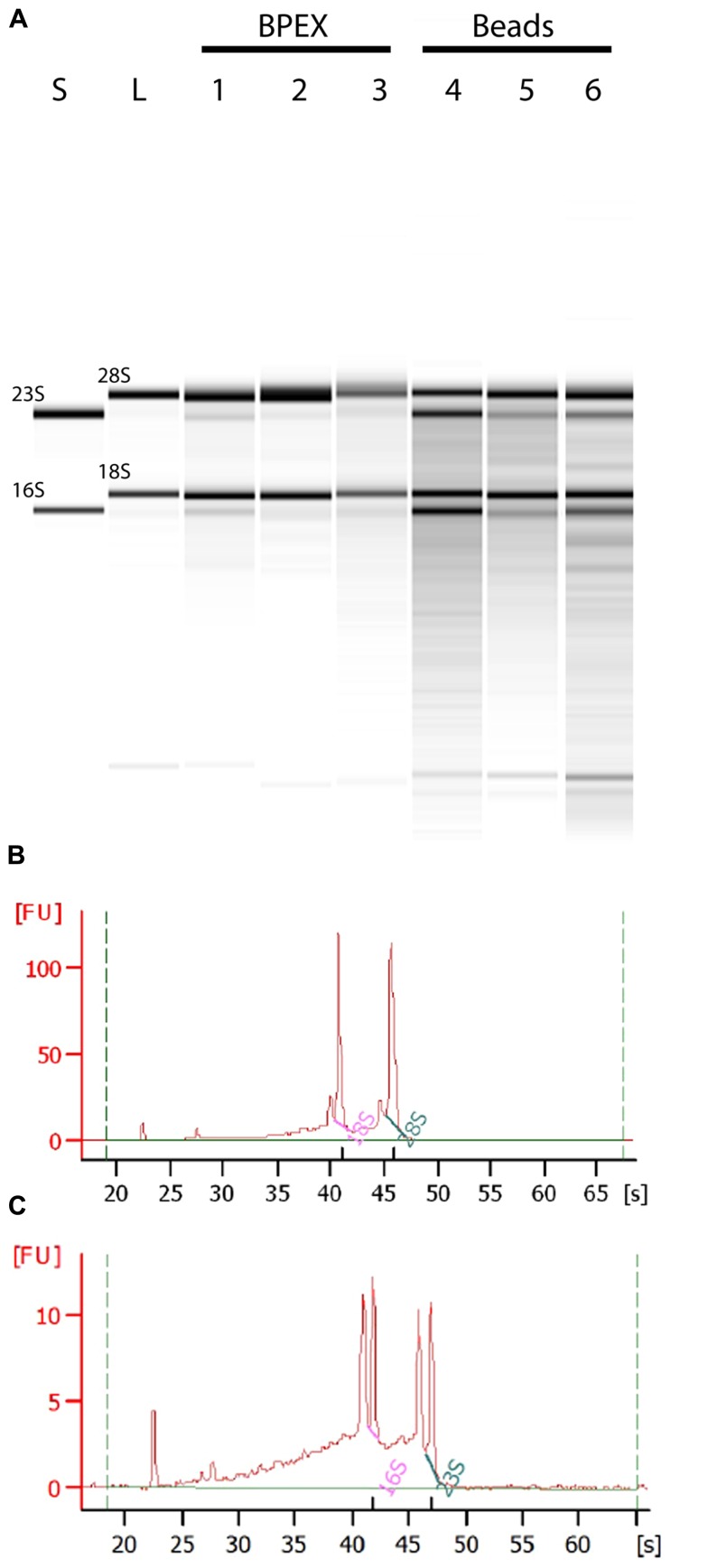
**Total RNA from plant root extractions by BPEX and Bead/SDS/phenol methods**. Intact plant roots were suspended in 20 ml of bacterial inoculum (OD_600_ of 0.2) for 2 h at 18°C. Total RNA was extracted using either the BPEX or the Bead/SDS/phenol method and RNA quality was assessed by spectroscopy on a Bioanalyser 2100 machine (Agilent). **(A)** A montage of an electropherograph of the total RNA levels following extraction of inoculated plant material. The lanes show RNA from an *in vitro* culture of *E. coli* O157:H7 isolate Sakai only (lane S); uninfected lettuce roots (lane L); *E. coli* O157:H7 Sakai infected lettuce roots (Lane 1, 306 ng μl^-^^1^; Lane 4, 119 ng μl^-^^1^; Lane 5, 195 ng μl^-^^1^); and *E. coli* O157:H7 Sakai infected spinach roots (Lane 2, 248 ng μl^-^^1^; Lane 3, 301 ng μl^-^^1^; Lane 6, 141 ng μl^-^^1^). Lanes 1, 2, and 3 show extraction using the BPEX method, and Lanes 4, 5, and 6 using the Bead method. The samples were run alongside a commercial RNA transcript ladder (0.2, 0.5, 1.0, 2.0, 4.0, and 6.0 kb, Agilent); the 16S and 23S (bacterial), and 18S and 28S (plant) *r*RNA bands are indicated. Electropherogram of *E. coli* O157:H7 Sakai infected lettuce roots extracted with **(B)** the BPEX method (sample derived from lane 1) or with **(C)** the novel Bead/SDS/phenol method (sample derived from lane 4). The uninfected spinach control was similar to lettuce (lane L) (not shown).

Use of the BPEX method typically yielded 3.6–9.6 μg of total RNA from 250 mg *E. coli* O157:H7-infected lettuce roots. Analysis of the total RNA showed strong bands corresponding with 18S and 28S *r*RNA of lettuce (**Figure 2A**: Lanes 1, 2, 3) with only very faint bacterial RNA corresponding to 16S and 23S *r*RNA in mixed samples (**Figure 2A**; Lanes 1, 2, 3). Examination of the electropherogram trace showed that the bacterial *r*RNA peaks were considerably smaller than the plant *r*RNA peaks, e.g., BPEX sample 1 (**Figure 2B**). The pre-dominance of plant *r*RNA in the BPEX-prepared samples indicated that plant *m*RNA would also dominate over bacterial transcripts. Therefore, to increase the levels of bacterial *m*RNA with a concomitant decrease in plant-derived RNA, a modified method was designed. The aim was threefold: (i) to reduce the number of plants required per extraction; (ii) to reduce the carry-over of plant tissue in the sample; and (iii) incorporate a lysis step which targeted bacterial cells.

### DEVELOPMENT OF THE BEAD/SDS/PHENOL PROTOCOL

Physical disruption of tissues and cells using inert beads has been used for nucleic acid extraction from filamentous fungi (Leite et al., 2012), microalgae (Kim et al., 2012), soil and sludge samples (Griffiths et al., 2000), and in commercial kits. We postulated that this method of disruption may also aid in bacterial RNA extraction and used a combination of 1 mm glass beads and 0.1 mm silica beads for sample lysis. Since tissue can become over-heated during agitation, which leads to nucleic acid degradation, the samples were “rested” for 30 s on ice between the three treatment cycles.

Bacterial cells were specifically targeted for lysis in an attempt to reduce plant-derived nucleic acid contamination, with the inclusion of lysozyme in the extraction buffer. However, a combination of the hot SDS/phenol extraction protocol described previously (Jahn et al., 2008) with the bead beating step resulted in very low RNA yields; not greater than 0.5 μg. It was possible that contaminants or inhibitors from the plant material, such as polysaccharides, phenolic compounds, and secondary metabolites deleteriously affected RNA recovery. Therefore, the protocol was modified to include additional steps after the initial lysis and the incorporation of PEG and CTAB/NaCl treatments, followed by LiCl precipitation (**Figure 1**). The inclusion of high-molecular-weight PEG in the extraction protocol promotes the removal of polysaccharides and phenolic compounds from plant tissues that bind to or co-precipitate with RNA (Gehrig et al., 2000). CTAB is a detergent that acts to separate polysaccharides from nucleic acids (Chang et al., 1993; Jaakola et al., 2001). Lithium chloride is inefficient at precipitating DNA, proteins, or carbohydrates, and thus improves RNA yield and purity compared to other nucleic acid precipitation methods, i.e., ethanol and sodium acetate (Barlow et al., 1963; Cathala et al., 1983).

### ANALYSIS OF RNA YIELDS

A combination of physical disruption using beads together with the chemical treatments (**Figure 1**) resulted in total RNA concentrations that averaged 5.1 μg (range from 1.5 to 8.9 μg) from 250 mg of infected root tissue. Assessment of the RNA showed a substantial increase in bacterial-derived 16S and 23S *r*RNA in bead-treated samples compared to BPEX samples (**Figure 2A** – compare lanes 4–6 with lanes 1–3). The optimized method increased the bacterial *r*RNA to levels that were equivalent or close to plant *r*RNA (**Figure 2C**).

### QUANTITATIVE REVERSE TRANSCRIPTASE PCR (qPCR) ANALYSIS

Bacterial gene expression was quantified from the samples obtained using the optimized method, by carrying out qPCR. The *gyrB* gene was selected as a housekeeping gene that was expected to be expressed under the conditions tested and expression from the root-derived samples was compared to samples obtained from bacteria grown under *in vitro* conditions. The amount of *gyrB* transcript was found to be similar between both lettuce roots and *in vitro* samples (**Table 1**), indicating similar copy numbers and stable *gyrB* expression under *in vitro* conditions and in plant extracts. It is of note that the amount of detectable *gyrB* was lower in the infected spinach root extracts compared to lettuce roots (higher *Ct* value for *gyrB*).

**Table 1:** Comparison of Ct values from *E. coli* O157:H7 isolate Sakai for an *in vitro* culture and for infected plant roots.

Sample	Average Ct ± SD
Sakai culture	23.435 ± 0.415
Sakai + lettuce roots	23.525 ± 0.295
Sakai + spinach roots	26.997 ± 0.527

*In brief, plant roots were suspended in 20 ml bacterial inoculum (OD_600_ of 0.2) for 2 h at 18°C, washed vigorously, and RNA prepared. The bacteria-only culture was suspended in 20 ml 1× PBS for 2 h at 18°C. The *gyrB* gene was quantified using a StepOnePlus PCR machine (Applied Biosystems). The values show the average of six samples from two independent experiments.*

### QUANTIFICATION OF BACTERIA

To quantify the bacteria present in the samples, the average number of *E. coli* O157:H7 (Sakai) cells present in a 250 mg root sample (typically from 7 to 8 roots) was determined. The experimental set-up was as described for RNA extraction, although the roots were washed with PBS instead of the initial step of *m*RNA preservation in the ice-cold phenol/ethanol mixture. On average, 5.6 × 10^7^ cfu of *E. coli* O157:H7 (Sakai) were present in each lettuce sample. The values for spinach roots were similar, which represent between 10 and 15% of the initial inoculum used for infection. These figures are equivalent to the limit of bacterial gene detection reported by Schenk et al. (2008) of ~ 5 10^7^ cfu/sample derived from 20 leaf disks.

## DISCUSSION

The isolation of total RNA from plant material is well defined for leaves, but less so for studies involving the roots or rhizosphere. The majority of reports focus on analysis of gene expression for phytopathogens and the number of examples describing the extraction of food-borne pathogen RNA from plants is limited. Here, we describe optimization of a method that resulted in high-quality bacterial *m*RNA from the infected roots of fresh produce plants: lettuce and spinach.

Reported investigations of gene expression of enteric bacteria, such as *Salmonella enterica* (Goudeau et al., 2013), *E. coli* K-12 and *E. coli* O157:H7 (Kyle et al., 2010; Fink et al., 2012) used large quantities of plant material, e.g., 100 g of leaf tissue coupled with high concentrations of bacteria, approx. 10^8^ cfu per sample. Samples were processed to separate bacteria from plant tissue using physical methods, such as a Stomacher (Kyle et al., 2010; Goudeau et al., 2013) or by shaking (Fink et al., 2012) followed by filtration to remove plant debris. RNA was prepared either using commercial kits (Kyle et al., 2010; Goudeau et al., 2013) or a hot-phenol method (Fink et al., 2012). Some of these studies investigated gene expression on post-harvest material, using pre-packaged commercial leaves rather than propagating plants, where it is possible to obtain the large amount of material required. In contrast, investigation of bacteria gene expression on living plant roots presents challenges in obtaining similar weights of material. A hydroponic system for high-throughput propagation of lettuce plants was described recently (Hou et al., 2012, 2013), where sterilized seeds were germinated in 96-well pipette tip boxes. Whole transcriptome analysis of *E. coli* was examined three days after inoculation with the plant roots, although the report does not state the weight of material required for RNA extraction. The main difference in our method was the amount of plant material required and the time at which the samples were taken. For example, our method allows for gene expression analysis after a short time of interaction, rather than after several days of colonization where the bacterial numbers are likely to have increased, dependent on the inoculation time and plant-bacterial system investigated.

The optimized method uses a combination of bead-beating, SDS lysis, phenol extraction, and CTAB purification to extract high-quality bacterial RNA from as little as 250–300 mg infected roots. Optimization has been carried out for two fresh produce plant species that have previously been associated with large-scale outbreaks of food-borne pathogens. It was interesting to note that the efficiency of recovery was lower for spinach root compared to lettuce, although the numbers of bacteria recovered from the plant tissue were similar. Since *E. coli* O157:H7 Sakai adheres to lettuce and spinach roots in comparable numbers (Wright et al., 2013), we anticipated similar levels of *gyrB* transcript. The reduction in level may be as a result of plant-associated inhibitors in spinach roots, or because of different plant-derived environmental cues acting on *gyrB* expression. Therefore, we suggest that the method needs to be validated if it is adopted for other plant species. Verification of the technique using qPCR shows that this method could be applied to food safety settings, e.g., for detection of food-borne pathogens in fresh produce. It may be possible to couple this technique to standard methods, which would enable examination of viability and expression of genes of interest, e.g., toxin genes. Furthermore, it facilitates analysis of bacterial gene expression *in planta* for not only food-borne pathogens but also other plant-associated bacteria, providing an insight into the adaptive processes that underpin host–microbe interactions.

## AUTHOR CONTRIBUTIONS

Al authors were involved in the design of the work and interpretation of data; Ashleigh Holmes and Louise Birse were involved in data acquisition and analysis; Ashleigh Holmes, Louise Birse, and Nicola J. Holden drafted the work; all authors approved the final version.

## Conflict of Interest Statement

The authors declare that the research was conducted in the absence of any commercial or financial relationships that could be construed as a potential conflict of interest.

## References

[B1] An S.-Q. Febrer M. Mccarthy Y. Tang D.-J. Clissold L. Kaithakottil G. (2013). High-resolution transcriptional analysis of the regulatory influence of cell-to-cell signalling reveals novel genes that contribute to Xanthomonas phytopathogenesis. *Mol. Microbiol.* 88 1058–1069 10.1111/mmi.12229 23617851 PMC3744752

[B2] Barak J. D. Schroeder B. K. (2012). Interrelationships of food safety and plant pathology: the life cycle of human pathogens on plants. *Annu. Rev. Phytopathol.* 50 241–266 10.1146/annurev-phyto-081211-172936 22656644

[B3] Barlow J. J. Mathias A. P. Williamson R. Gammack D. B. (1963). A simple method for the quantitative isolation of undegraded high molecular weight ribonucleic acid. *Biochem. Biophys. Res. Commun.* 13 61–66 10.1016/0006-291X(63)90163-3 14069514

[B4] Brandl M. T. (2006). Fitness of human enteric pathogens on plants and implications for food safety. *Annu. Rev. Phytopathol.* 44 367–392 10.1146/annurev.phyto.44.070505.143359 16704355

[B5] Buchholz U. Bernard H. Werber D. Bohmer M. M. Remschmidt C. Wilking H. (2011). German outbreak of *Escherichia coli* O104:H4 associated with sprouts. *N. Engl. J. Med*. 365 1763–1770 10.1056/NEJMoa1106482 22029753

[B6] Cathala G. Savouret J. F. Mendez B. West B. L. Karin M. Martial J. A. (1983). A method for isolation of intact, translationally active ribonucleic acid. *DNA* 2 329–335 10.1089/dna.1983.2.329 6198133

[B7] Chang S. Puryear J. Cairney J. (1993). A simple and efficient method for isolating RNA from pine trees. *Plant Mol. Biol. Rep.* 11 113–116 10.1007/BF02670468

[B8] Cooley M. Carychao D. Crawford-Miksza L. Jay M. T. Myers C. Rose C. (2007). Incidence and tracking of *Escherichia coli* O157:H7 in a major produce production region in California. *PLoS ONE* 2:e1159 10.1371/journal.pone.0001159 PMC2174234 18174909

[B9] Fink R. C. Black E. P. Hou Z. Sugawara M. Sadowsky M. J. Diez-Gonzalez F. (2012). Transcriptional responses of *Escherichia coli* K-12 and O157:H7 associated with lettuce leaves. *Appl. Environ. Microbiol.* 78 1752–1764 10.1128/aem.07454-11 22247152 PMC3298177

[B10] Fislage R. Berceanu M. Humboldt Y. Wendt M. Oberender H. (1997). Primer design for a prokaryotic differential display RT-PCR. *Nucl. Acids Res.* 25 1830–1835 10.1093/nar/25.9.1830 9108168 PMC146661

[B11] Gehrig H. H. Winter K. Cushman J. Borland A. Taybi T. (2000). An improved RNA isolation method for succulent plant species rich in polyphenols and polysaccharides. *Plant Mol. Biol. Rep.* 18 369–376 10.1007/BF02825065

[B12] Goudeau D. M. Parker C. T. Zhou Y. Sela S. Kroupitski Y. Brandl M. T. (2013). The Salmonella transcriptome in lettuce and cilantro soft rot reveals a niche overlap with the animal host intestine. *Appl. Environ. Microbiol.* 79 250–262 10.1128/aem.02290-12 23104408 PMC3536078

[B13] Griffiths R. I. Whiteley A. S. O’Donnell A. G. Bailey M. J. (2000). Rapid method for coextraction of DNA and RNA from natural environments for analysis of ribosomal DNA- and rRNA-based microbial community composition. *Appl. Environ. Microbiol.* 66 5488–5491 10.1128/AEM.66.12.5488-5491.2000 11097934 PMC92488

[B14] Hayashi T. Makino K. Ohnishi M. Kurokawa K. Ishii K. Yokoyama K. (2001). Complete genome sequence of enterohemorrhagic *Escherichia coli* O157:H7 and genomic comparison with a laboratory strain K-12. *DNA Res.* 8 11–22 10.1093/dnares/8.1.11 11258796

[B15] Hernandez-Morales A. De La Torre-Zavala S. Ibarra-Laclette E. Hernandez-Flores J. Jofre-Garfias A. Martinez-Antonio A. (2009). Transcriptional profile of *Pseudomonas syringae* pv. phaseolicola *NPS*3121 in response to tissue extracts from a susceptible *Phaseolus vulgaris* L. cultivar. *BMC Microbiol.* 9:257 10.1186/1471-2180-9-257 PMC2803797 20003402

[B16] Holden N. Pritchard L. Toth I. (2009). Colonization outwith the colon: plants as an alternative environmental reservoir for human pathogenic enterobacteria. *FEMS Microbiol. Rev.* 33 689–703 10.1111/j.1574-6976.2008.00153.x 19076238

[B17] Hou Z. Fink R. C. Black E. Sugawara M. Zhang Z. Diez-Gonzalez F. (2012). Gene expression profiling of *Escherichia coli* in response to interactions with the lettuce rhizosphere. *J. Appl. Microbiol.* 113 1076–1086 10.1111/j.1365-2672.2012.05412.x 22830299

[B18] Hou Z. Fink R. C. Sugawara M. Diez-Gonzalez F. Sadowsky M. J. (2013). Transcriptional and functional responses of *Escherichia coli* O157:H7 growing in the lettuce rhizoplane. *Food Microbiol.* 35 136–142 10.1016/j.fm.2013.03.002 23664265

[B19] Jaakola L. Pirttila A. M. Halonen M. Hohtola A. (2001). Isolation of high quality RNA from bilberry (*Vaccinium myrtillus* L.) fruit. *Mol. Biotechnol.* 19 201–203 10.1385/mb:19:2:201 11725489

[B20] Jahn C. E. Charkowski A. O. Willis D. K. (2008). Evaluation of isolation methods and RNA integrity for bacterial RNA quantitation. *J. Microbiol. Methods* 75 318–324 10.1016/j.mimet.2008.07.004 18674572

[B21] Kim B.-H. Ramanan R. Cho D.-H. Choi G.-G. La H.-J. Ahn C.-Y. (2012). Simple, rapid and cost-effective method for high quality nucleic acids extraction from different strains of *Botryococcus braunii*. *PLoS ONE* 7:e37770 10.1371/journal.pone.0037770 PMC3360624 22662217

[B22] Kyle J. L. Parker C. T. Goudeau D. Brandl M. T. (2010). Transcriptome analysis of *Escherichia coli* O157:H7 exposed to lysates of lettuce leaves. *Appl. Environ. Microbiol.* 76 1375–1387 10.1128/AEM.02461-09 20061451 PMC2832375

[B23] Leite G. M. Magan N. Medina A. (2012). Comparison of different bead-beating RNA extraction strategies: an optimized method for filamentous fungi. *J. Microbiol. Methods* 88 413–418 10.1016/j.mimet.2012.01.011 22289387

[B24] Mark G. L. Dow J. M. Kiely P. D. Higgins H. Haynes J. Baysse C. (2005). Transcriptome profiling of bacterial responses to root exudates identifies genes involved in microbe-plant interactions. *Proc. Natl. Acad. Sci. U.S.A.* 102 17454–17459 10.1073/pnas.0506407102 16301542 PMC1297666

[B25] Matilla M. A. Espinosa-Urgel M. Rodriguez-Herva J. J. Ramos J. L. Ramos-Gonzalez M. I. (2007). Genomic analysis reveals the major driving forces of bacterial life in the rhizosphere. *Genome Biol.* 8 R179 10.1186/gb-2007-8-9-r179 PMC2375017 17784941

[B26] Neidhardt F. C. Bloch P. L. Smith D. F. (1974). Culture medium for enterobacteria. *J. Bacteriol.* 119 736–747 4604283 10.1128/jb.119.3.736-747.1974 PMC245675

[B27] Rossez Y. Holmes A. Wolfson E. B. Gally D. L. Mahajan A. Pedersen H. L. (2013). Flagella interact with ionic plant lipids to mediate adherence of pathogenic *Escherichia coli* to fresh produce plants. *Environ. Microbiol.* 10.1111/1462-2920.12315 [Epub ahead of print] 24148193

[B28] Roy D. Panchal S. Rosa B. A. Melotto M. (2013). *Escherichia coli* O157:H7 induces stronger plant immunity than *Salmonella enterica* Typhimurium SL1344. *Phytopathology* 103 326–332 10.1094/PHYTO-09-12-0230-FI 23301812 PMC3982233

[B29] Schenk A. Weingart H. Ullrich M. S. (2008). Extraction of high-quality bacterial RNA from infected leaf tissue for bacterial in planta gene expression analysis by multiplexed fluorescent Northern hybridization. *Mol. Plant Pathol.* 9 227–235 10.1111/j.1364-3703.2007.00452.x 18705854 PMC6640379

[B30] Schikora A. Carreri A. Charpentier E. Hirt H. (2008). The dark side of the salad: *Salmonella typhimurium* overcomes the innate immune response of *Arabidopsis thaliana* and shows an endopathogenic lifestyle. *PLoS ONE* 3:e2279 10.1371/journal.pone.0002279 PMC2386236 18509467

[B31] Shidore T. Dinse T. Öhrlein J. Becker A. Reinhold-Hurek B. (2012). Transcriptomic analysis of responses to exudates reveal genes required for rhizosphere competence of the endophyte *Azoarcus sp*. strain BH72. *Environ. Microbiol.* 14 2775–2787 10.1111/j.1462-2920.2012.02777.x 22616609

[B32] Soto-Suarez M. Bernal D. Gonzalez C. Szurek B. Guyot R. Tohme J. (2010). In planta gene expression analysis of *Xanthomonas oryzae* pathovar oryzae, African strain MAI1. *BMC Microbiol*. 10:170 10.1186/1471-2180-10-170 PMC2893596 20540733

[B33] Thilmony R. Underwood W. He S. Y. (2006). Genome-wide transcriptional analysis of the *Arabidopsis thaliana* interaction with the plant pathogen *Pseudomonas syringae* pv. *tomato DC*3000 and the human pathogen *Escherichia coli* O157:H7. *Plant J*. 46 34–53 10.1111/j.1365-313X.2006.02725.x 16553894

[B34] Wright K. M. Chapman S. Mcgeachy K. Humphris S. Campbell E. Toth I. K. (2013). The endophytic lifestyle of *Escherichia coli* O157:H7: quantification and internal localization in roots. *Phytopathology* 103 333–340 10.1094/PHYTO-08-12-0209-FI 23506361

[B35] Zyśko A. Sanguin H. Hayes A. Wardleworth L. Zeef L. H. Sim A. (2012). Transcriptional response of *Pseudomonas aeruginosa* to a phosphate-deficient *Lolium perenne* rhizosphere. *Plant Soil* 359 25–44 10.1007/s11104-011-1060-z

